# Institutional Notes and News

**Published:** 1910-07-09

**Authors:** 


### Institutional Notes and News.

**Thetford Cottage Hospital.**

The Thetford Cottage Hospital, Norfolk, has finished its year with a satisfactory balance-sheet, £270 having been received and £249 having been spent. The legacy of £1,000 from the late Mr. Vavasseur has been received and invested. An interesting question has been raised as to the advisability of admitting paying patients, but as their admittance involves an alteration in the rules of the hospital, which requires a fortnight's notice before it can be discussed, the committee of management are to consider it and report whatever the result of their consideration may be. It is satisfactory to note that the small hospitals of Norfolk are to share with the big hospitals the sum it is hoped will be raised by the movement for commemorating King Edward. The Mayor of Thetford is to take up the matter and see if an endowment of £100 cannot be raised as a local memorial also. It should be added that forty-one cases were treated in the course of the year.

**Maitland Sanatorium.**

The new buildings of the Maitland Sanatorium at Peppard Common, near Reading, were opened by the Bishop of Oxford on June 30. The company included Professor Osler, Dr. Esther Carling, Dr. Florence Armitage, and Dr. G. Stewart Abram. The chairman (the Hon. W. F. D. Smith) said that they had received for the alterations and additions £3,469. They had spent £3,080, and there were works in hand which would cost £900. That left a balance of £511 to make up, and he hoped the debt would soon be liquidated. The Bishop of Oxford heartily commended for their reading the speech made by Mr. John Burns at the Whitechapel Exhibition last year, which showed in a most valuable and suggestive way the multitude of channels through which directly or indirectly tuberculosis was hindering all our national welfare. Professor Osler said that the campaign against consumption was by far the most hopeful campaign in which they were engaged to-day. In the course of a few generations the disease would be stamped out. Mr. J. F. Mason, M.P., in proposing a vote of thanks to the chairman, promised to double whatever sum was collected in aid of the funds.

**Cumberland Infirmary.**

A scheme for the enlargement and improvement of the Cumberland Infirmary, at a cost of about £30,000, started in 1908, is almost completed. It provides a new west pavilion, containing on the ground floor a male surgical ward with 12 beds, and on the first floor a new children's ward with 24 cots; an entirely new kitchen block; a new nurses' home for 40 nurses; a remodelled and enlarged laundry, fitted with steam-worked machinery; the reorganisation of the heating and hot-water service; better facilities for the treatment of casualties; new ophthalmic and *x*-ray departments; several structural alterations to the old part of the infirmary; and a new carriage shelter for the use of the honorary medical staff. Of this comprehensive work there only remain unfinished the new west pavilion (which is being built) and the enlarged out-patient department. When the whole scheme is completed, the accommodation for in-patients will be raised from 102 to over 150. We hope that the money for maintaining the extended hospital will be raised as successfully as the new buildings have been.

**Swaffham Cottage Hospital.**

In spite of the success of the Pound Day, which was started for the first time last year at the Swaffham Cottage Hospital, Norfolk, there was a debt on the working expenses of £30 at the end of the year. This, added to the previous deficit, makes a total debt of £52. It is hoped, however, that the shilling fund, founded in memory of the late King, and the house-to-house visit which also is to be undertaken, will succeed in stemming the tide and in putting the hospital once more on a sound footing. Thirty patients were treated in the wards and twenty-eight persons attended at the out-patient department. One scarlet-fever case occurred, and the expense this involved is mentioned in the annual report as accounting for the sum by which the hospital's debt on the working expenses has increased lately. Mr. W. Nash, who seconded the adoption of the report at the recent annual meeting, said that the hospital really needed an income of £250 a year. Mr. J. A. Gould and Mr. J. O. Dennis have been re-elected Hon. Secretaries, and Mr. J. W. Page Hon. Treasurer.

**Albert Edward Infirmary, Wigan.**

Mr. James Wilson, in the unavoidable absence of the Mayor of Wigan, Mr. Wood, presided over the annual meeting of the Royal Albert Edward Infirmary, Wigan. The total income for the past year was £13,474 and the total expenditure £11,680; in other words, the income had gone up and the expenditure had gone down, nearly £2,000 being saved in spite of the increased work, and over £400 gained as compared with the previous year's revenue. We may note, too, that "the magnificent sum of £8,314" had been contributed by working people. There are two debts, however, one on the building fund of £7,807, a decrease of £1,311, and one on the maintenance fund of £4,038, a decrease of £375. As regards the patients, 11,980 was the total number of in-cases treated. Mr. James Wilson recalled the fact that in 1907 an appeal was issued for £25,000 to pay off their debts and to build a new out-patient department, to instal electric light, and to make various changes in the sleeping accommodation of the staff. That appeal has produced at present only £5,596. He pointed out that it is a common mistake to suppose that the Infirmary is more or less independent of subscriptions, the fact being that its endowments do not produce more than £2,500 a year, and that the patients cannot be attended to adequately with a smaller expenditure than £11,000 every twelve months. After eulogistic reference to Sir Henry Burdett's inspection and report had been made, Dr. Rees recommended the establishment of almoners to control the recommendations given in the out-patient department, because he believed the congestion often seen there was due to an improper class of person seeking treatment. He urged also that the Infirmary should be paid for treating children sent by the medical inspectors. Dr. Rees made an interesting criticism of the increased charges now made in the pay wards—three guineas instead of one guinea—on the ground that it made the hospital as inaccessible as a nursing home to those persons who, while unable to pay the fees of the latter, were willing to avoid a charitable institution by paying what they could afford. The guinea-a-week class was thus, he said, unprovided for. He knew, of course, that that sum did not cover the actual cost incurred. The Chairman, in reply, said that these points were being considered.

